# Supplementary material for: C. elegans chromosomes connect to centrosomes by anchoring into the spindle network
Source: Nat Commun. 2017 May 11;8:15288. doi: 10.1038/ncomms15288 (PMC5437269; doi:10.1038/ncomms15288)
Supplement: Supplementary Information — Supplementary Figures and Supplementary Tables [file ncomms15288-s1.pdf]

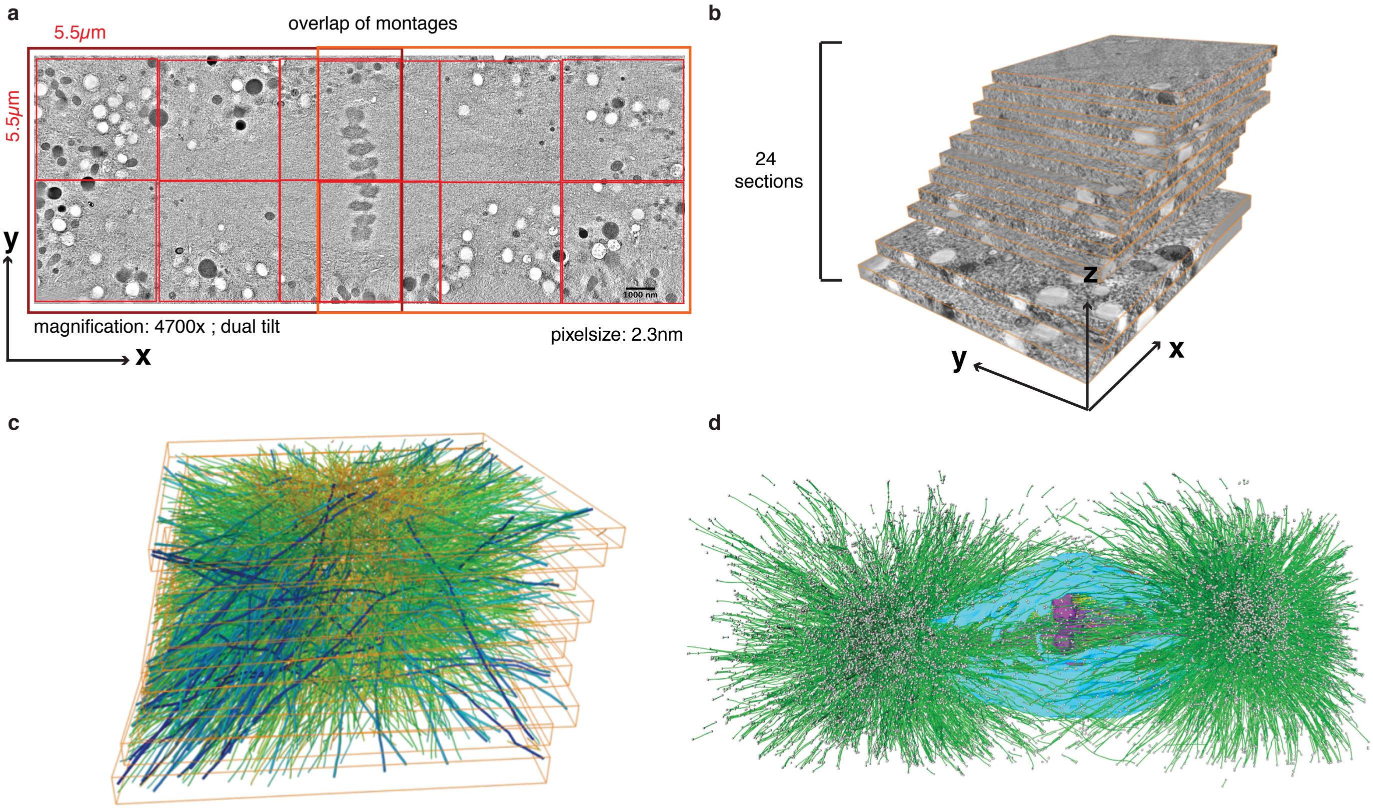

Supplementary Figure 1. Workflow of large-scale spindle reconstructions by 3D electron tomography

a, Two 2 x 3 montages (outlined in dark red, individual tomograms composing the montages are outlined in light red) are acquired and joined in X and Y to cover the entire area of the spindle. The size of a single tomogram, the magnification, and voxel size are indicated. The thickness of a section is 300 nm **b**, Approximately 25 consecutive sections have to be acquired to cover the spindle volume. **c**, Microtubules (green) are automatically traced and manually corrected using the AMIRA software. This software is also used to stitch the individual sections in z. **d**, Features like chromosomes (purple) or the nuclear envelope (light blue) are segmented manually.

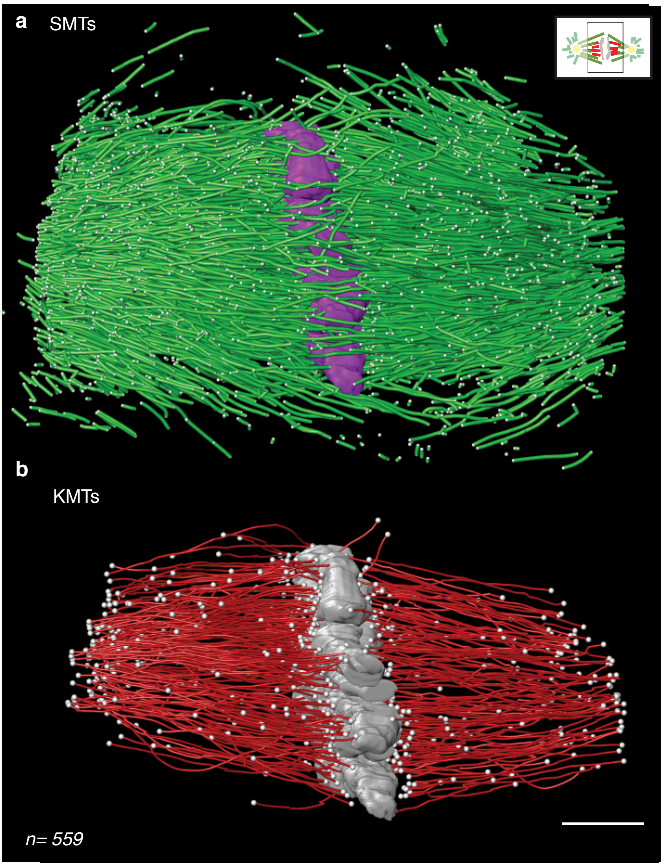

Supplementary Figure 2. Reconstruction of a central spindle in metaphase

a, Model of a metaphase spindle (Metaphase 3) covering the volume around the chromosomes. The region of the tomogram is indicated in the upper right corner. **b**, KMTs of the dataset as shown in a. The number of KMTs is indicated in the bottom left corner. Scale bar, 1 μm.

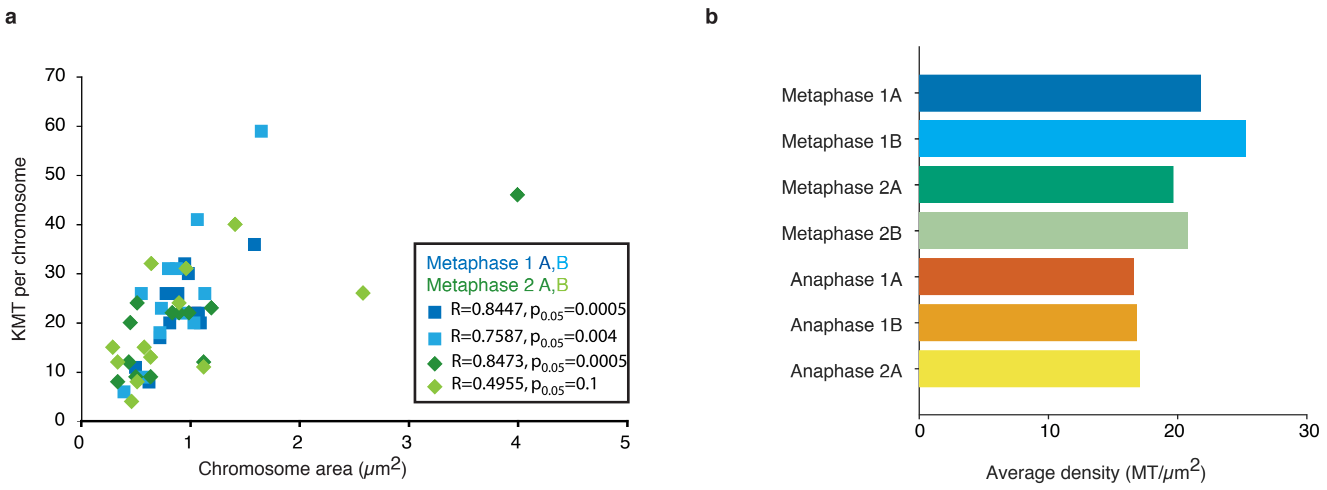

Supplementary Figure 3. KMT attachment correlates with chromosome area
a, Correlation of chromosome surface area and number of attached KMTs for two metaphase datasets. The Pearson's correlation coefficient is indicated. **b**, Density of KMT attachment sites on chromosomes in metaphase and anaphase averaged over all chromosomes.

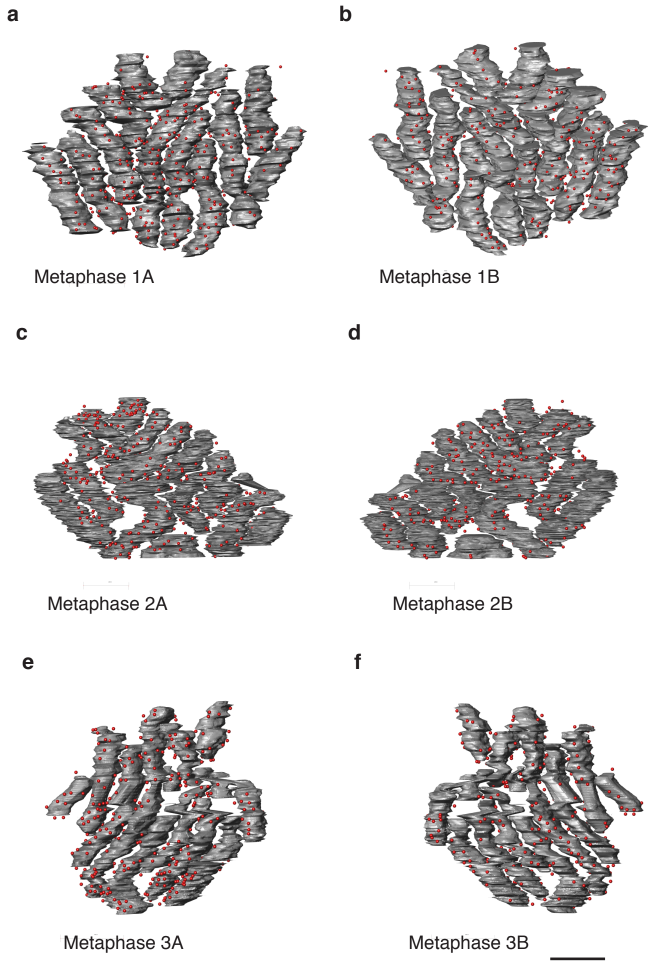

Supplementary Figure 4. KMT attachments sites on the chromosomes
End-on views of each metaphase plate as seen from both poles for the different data sets. **a**, **b**, Metaphase 1. **c**, **d**, Metaphase 2. **e**, **f**, Metaphase 3. Microtubule attachment to individual chromosomes from each pole is indicated by red dots. Scale bar, 1 μm.

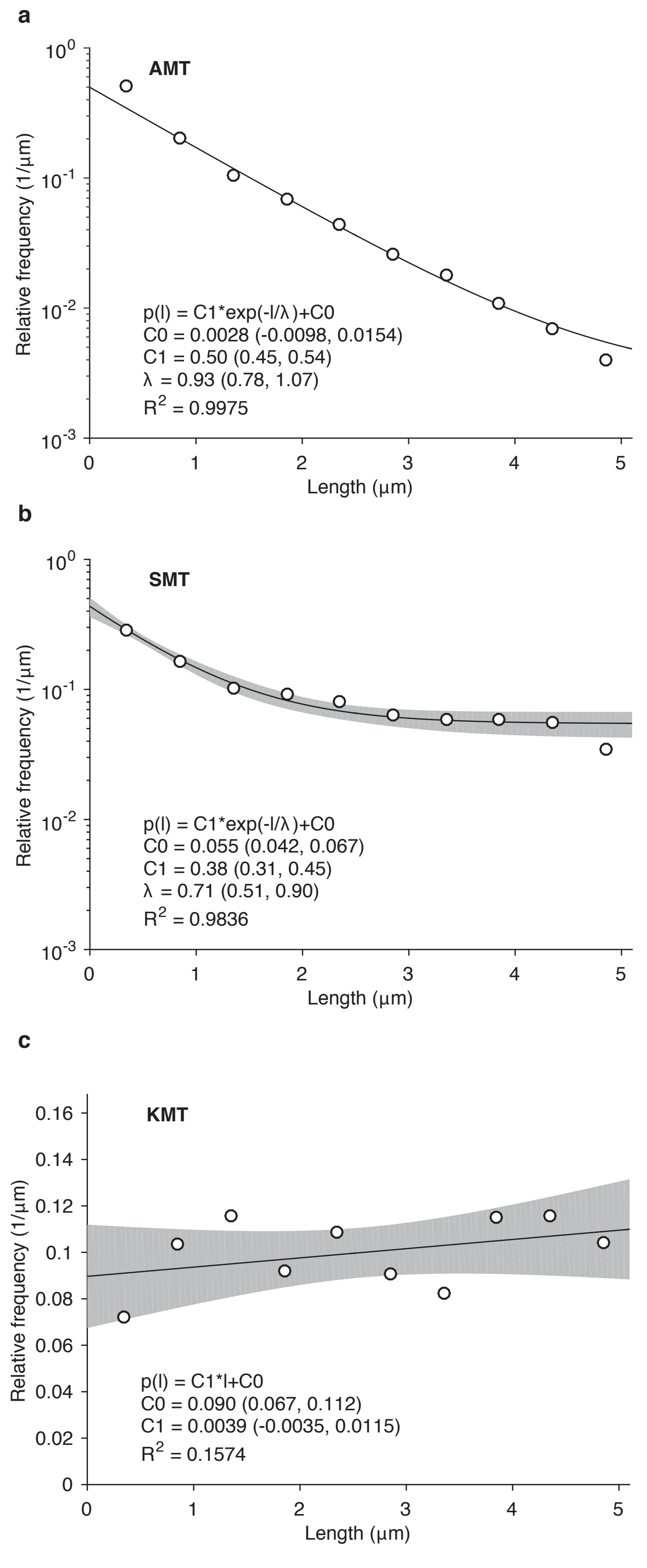

Supplementary Figure 5. Fitted histograms of the microtubule length distribution

a, Fit of the histogram of the AMT length distribution based on all five data sets to a single exponential with a constant. The gray shaded areas are the 95 % confidence intervals for the fitted function for single observations. **b**, Fit of the histogram of the SMT length distribution based on all five data sets to a single exponential plus a constant. **c**, Fit of the histogram of the KMT length distribution based on all five data sets to a linear function. The fitting parameters are indicated with the 95 % confidence intervals and the unadjusted coefficient of determination (R2) is provided.

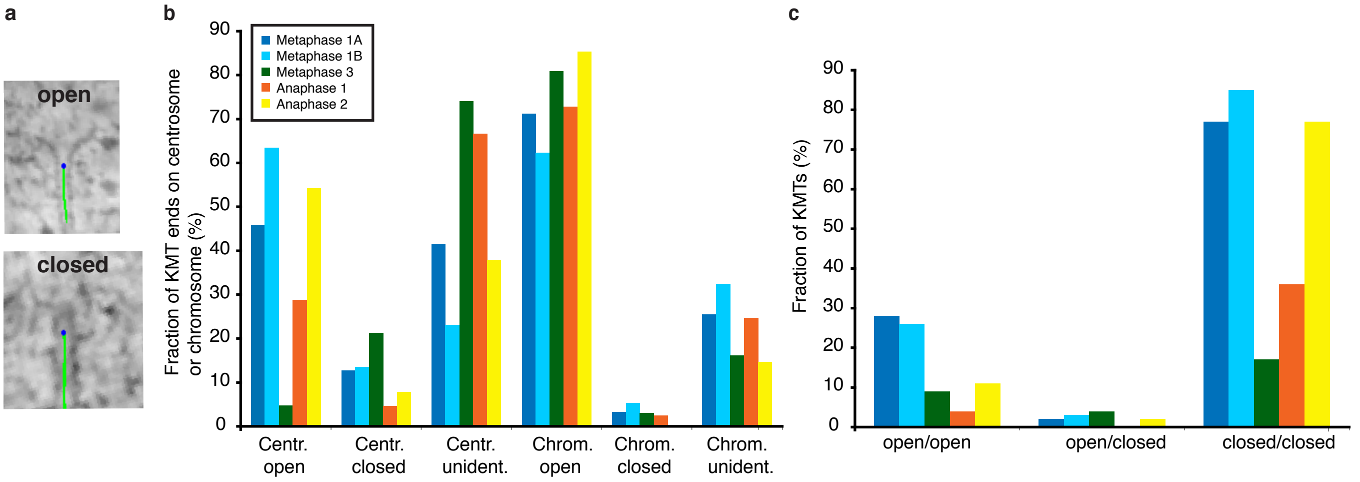

Supplementary Figure 6. Analysis of microtubule end conformation
a, Representative example for an open (upper panel) and closed microtubule end conformation (lower panel). **b**, Percentage of open, closed and unidentified KMT ends at the centrosomes and chromosomes in metaphase and anaphase. **c**, Percentage of conformations of both ends of individual KMTs in metaphase and anaphase.

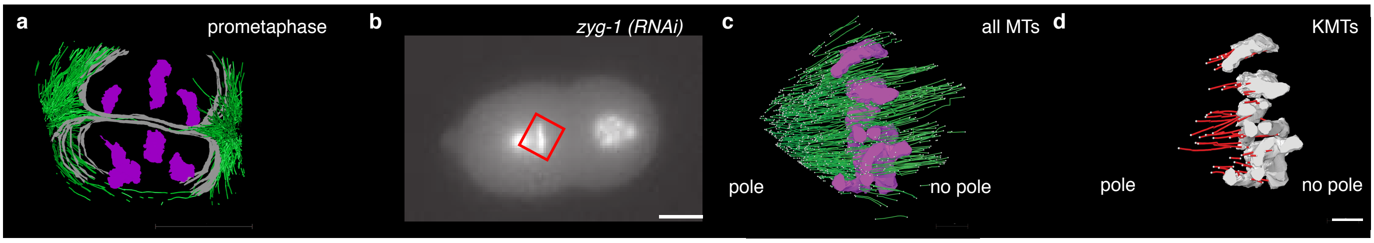

Supplementary Figure 7. Microtubules in early prometaphase and monopolar spindles
a, Model of chromosomes (magenta), nuclear envelope (grey) and microtubules (green) in a one-cell *C. elegans* embryo at early prometaphase. **b**, Two-cell *C. elegans* embryo after *zyg-1 (RNAi)* labeled with β-tubulin::GFP and Histone::GFP. Red box indicates the area of the tomogram Scale bar, 10 μm. **c**, Model of SMTs in three consecutive tomographic sections of a monopolar spindle as shown in b. **d**, Model of the KMTs as identified in c. Scale bar, 1 μm.

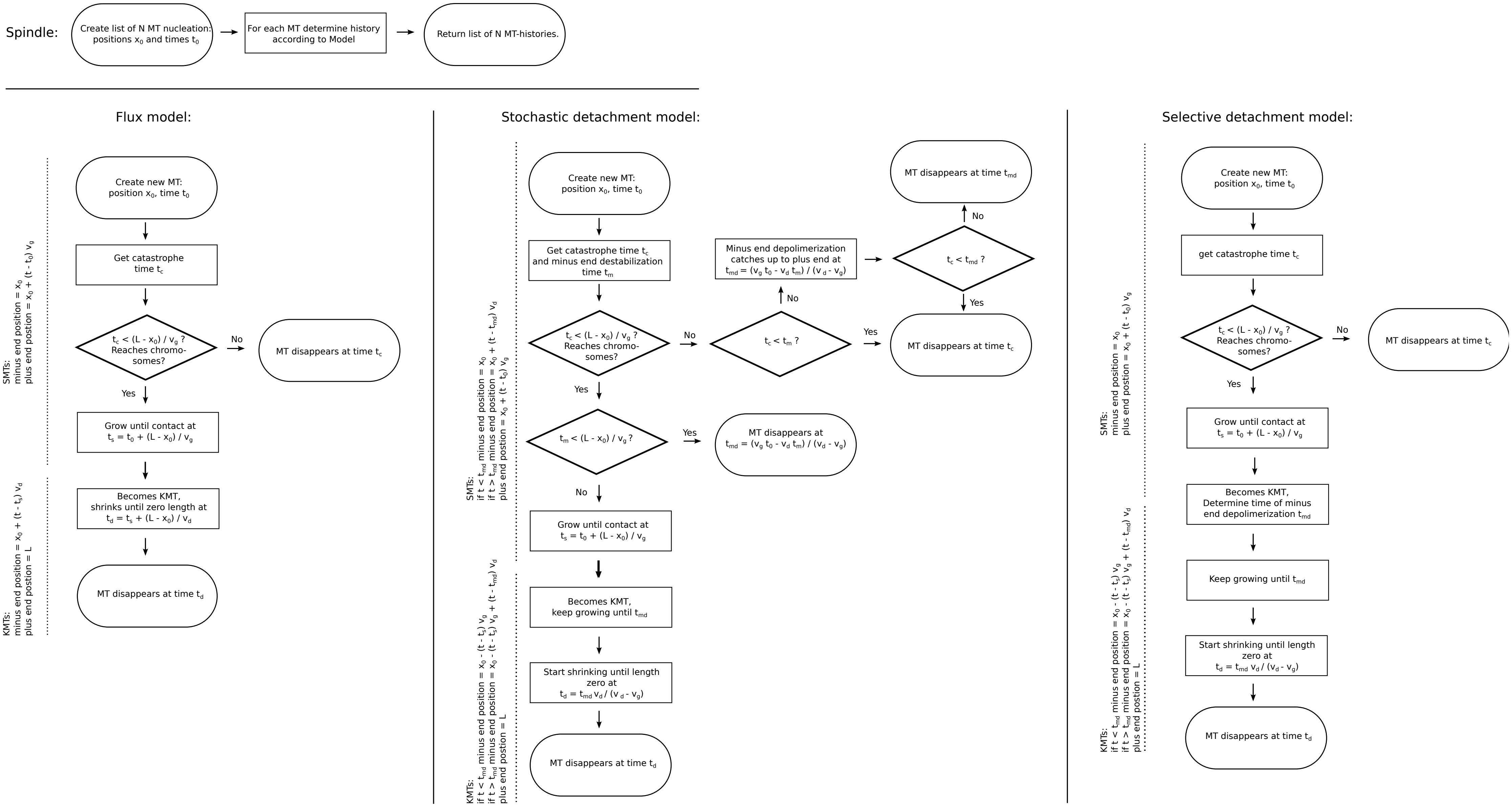

Supplementary Figure 8. Flowchart of the simulation process

Schematic flowchart depicting the different models in the context of a Gillespie algorithm.

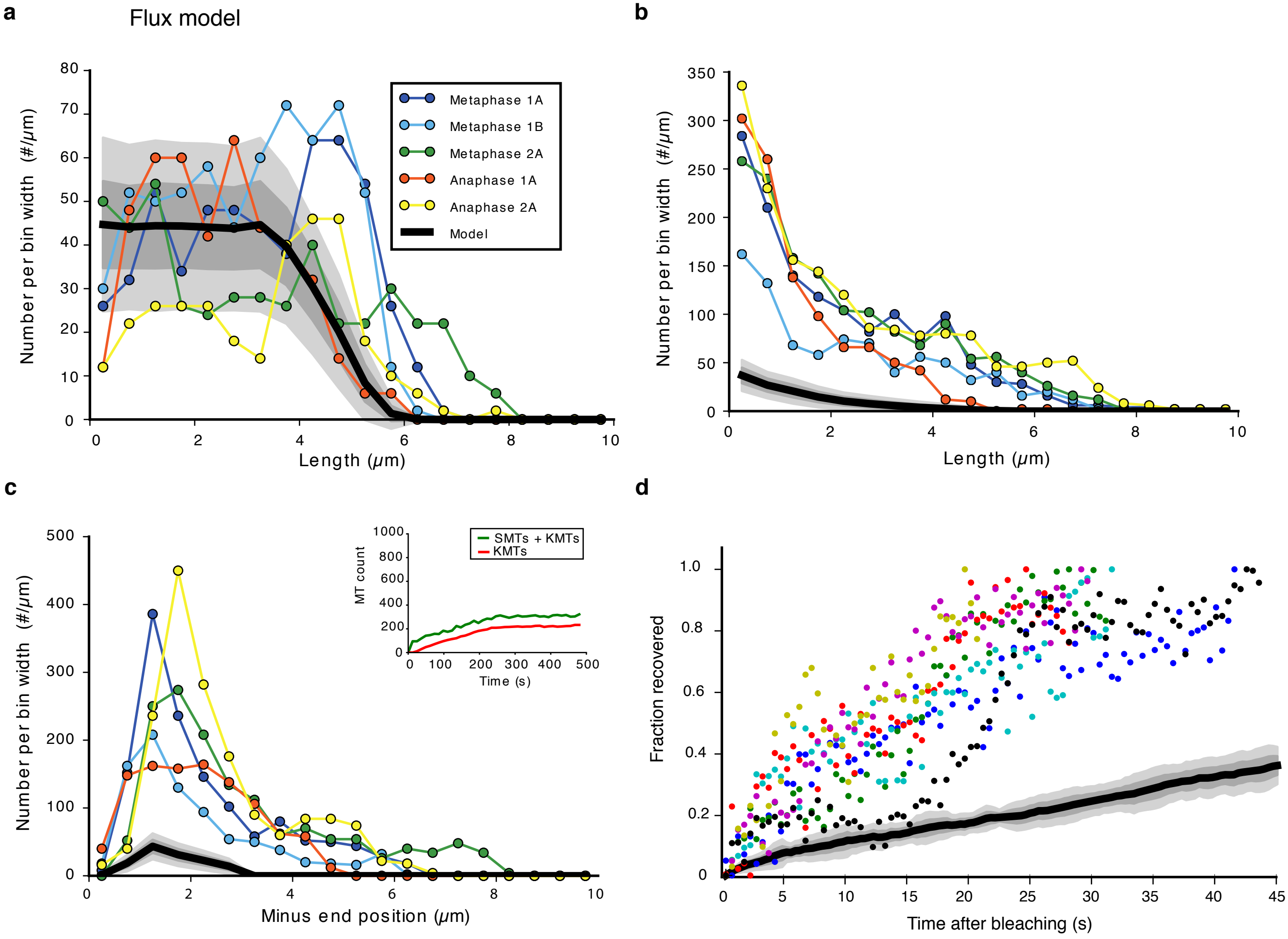

Supplementary Figure 9. Results from the stochastic microtubule flux model
a, KMT length distribution. **b**, SMT length distribution. **c**, SMT minus-end distribution. Inset shows the time-course of the total microtubule number (green) and KMT number (red) for a typical instance of the simulation with a depolymerisation velocity of $v_d = 0.03\ \mu m\ s^{-1}$
d, Comparison of experimental FRAP data on microtubule recovery (individual measurement are shown in different colours) with the simulated FRAP data based on the flux model.
For a, b, c, d we display the long expectation value of the model (solid black line) plus one (dark grey shaded region) and two (light grey shaded region) standard deviations.

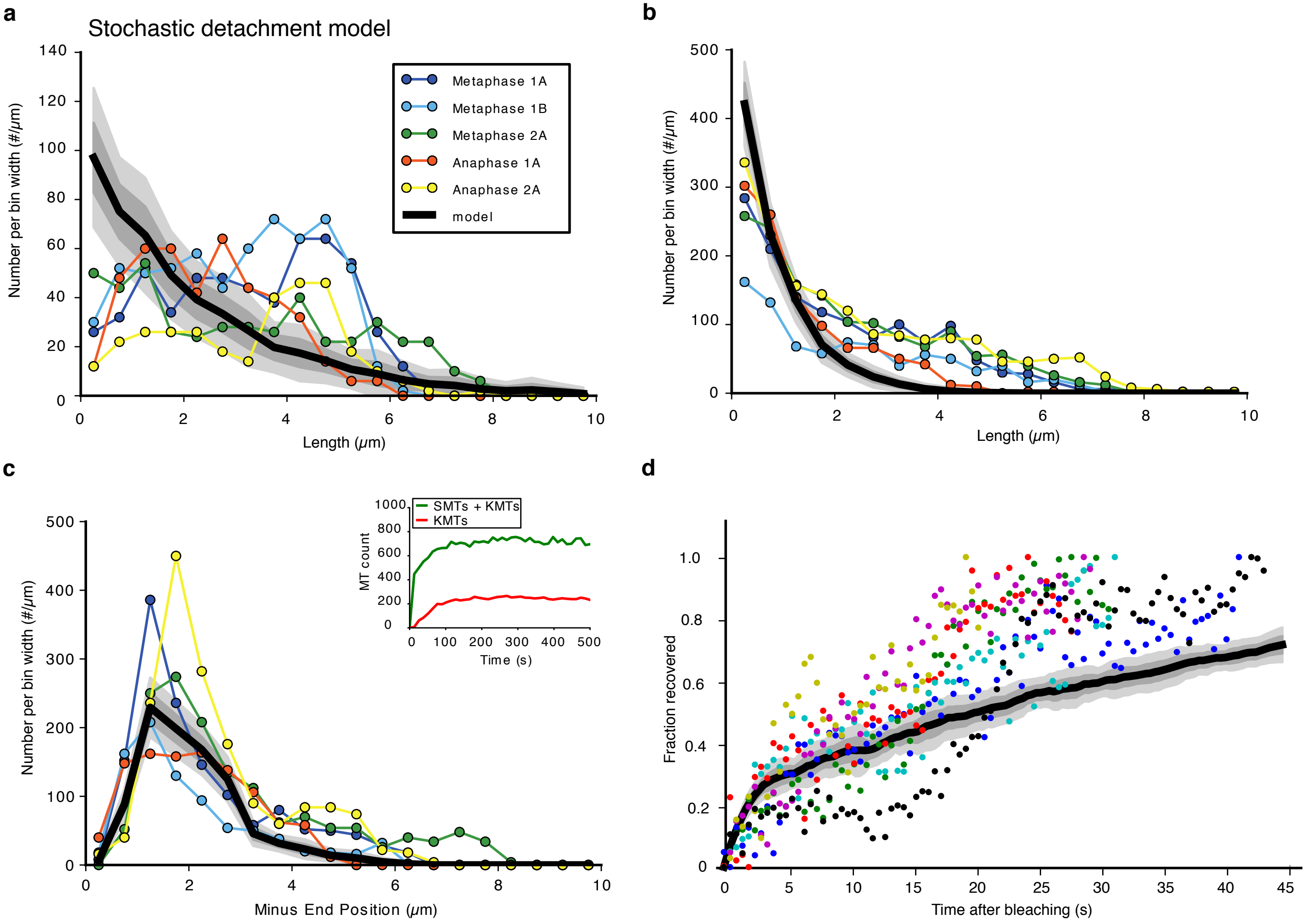

Supplementary Figure 10. Results from the stochastic microtubule detachment model
a, KMT length distribution. **b**, SMT length distribution. **c**, SMT minus end distribution. Inset shows the time-course of the total microtubule number (green) and KMT number (red) for a typical instance of the simulation with a depolymerisation velocity of $v_d = 0.45\ \mu m\ s^{-1}$ and a switching rate from growth to shrinkage of r = 0.1 Hz. **d**, Comparison of experimental FRAP data on microtubule recovery (individual measurement are shown in different colours) with the simulated FRAP data based on the stochastic detachment model. For a, b, c, d we display the long expectation value of the model (solid black line) plus one (dark grey shaded region) and two (light grey shaded region) standard deviations.

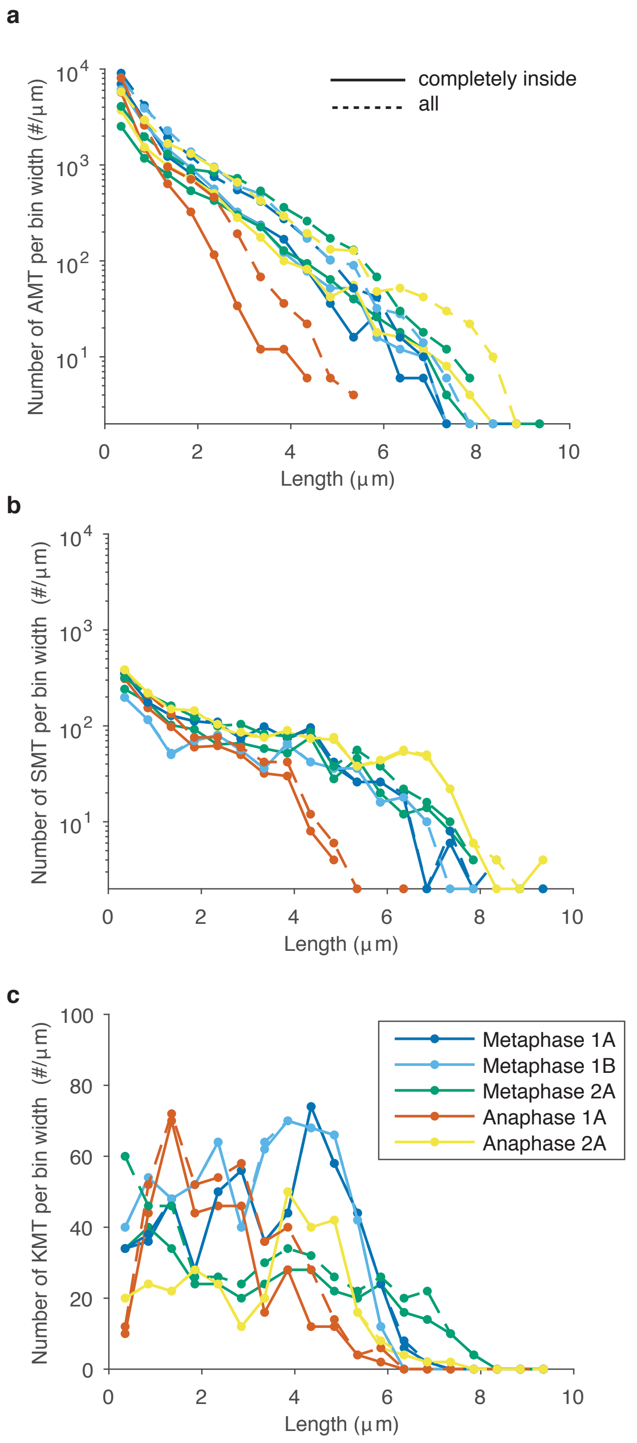

Supplementary Figure 11. Effect of the tomographic boundary on microtubule length distributions

a, Length distributions of all AMTs which are not touching the tomographic boundary and have both endpoints within the tomogram (solid line) and of all AMTs (dashed line). **b**, length distributions of all SMTs which are not touching the tomographic boundary and have both endpoints within the tomogram (solid line) and of all SMTs (dashed line). **c**, length distributions of all KMTs which are not touching the tomographic boundary and have both endpoints within the tomogram (solid line) and of all KMTs (dashed line).

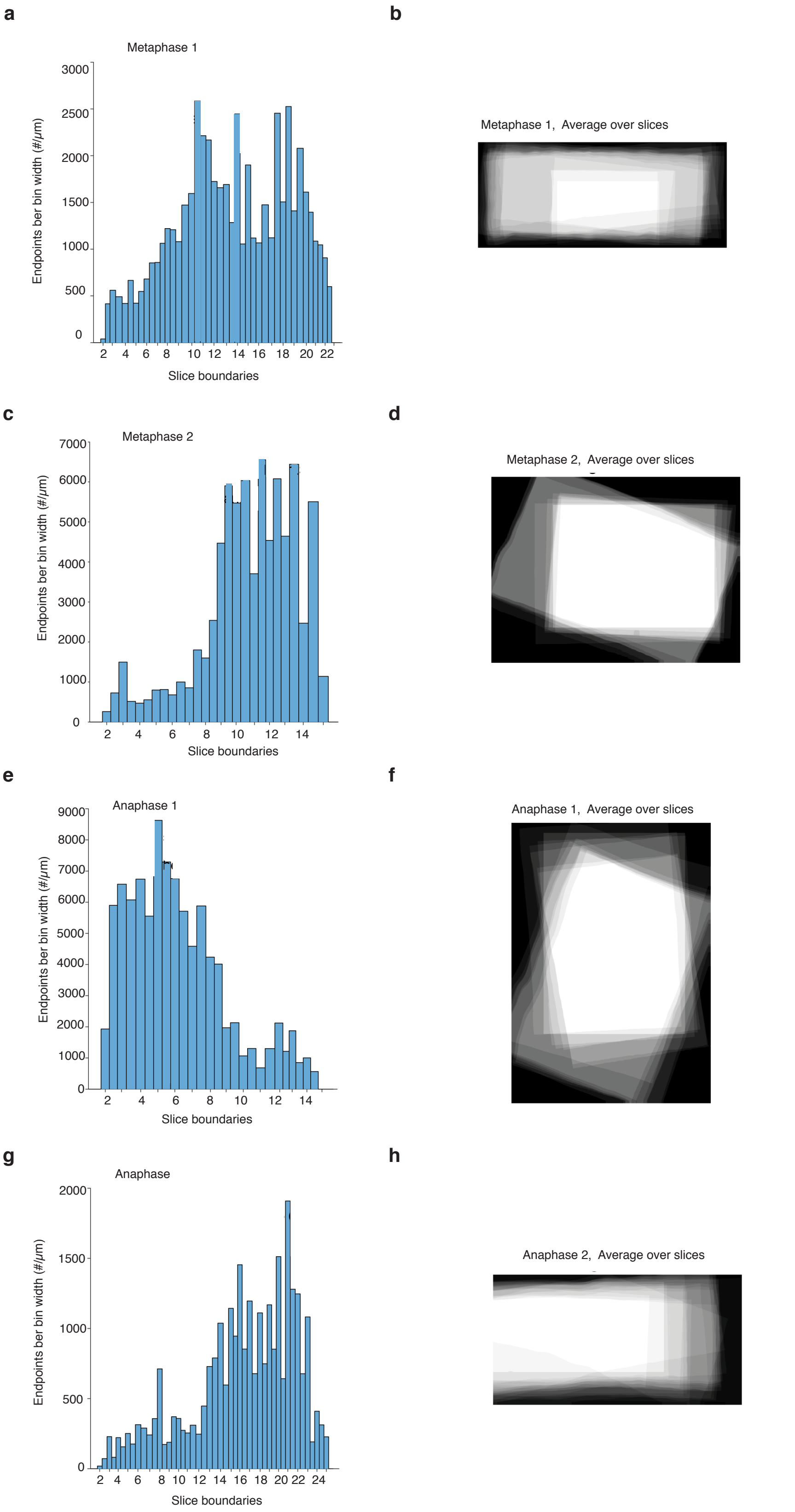

Supplementary Figure 12. Z-stack histograms of microtubule endpoints

a,**c**,**e**,**g**, Histograms of the number of microtubule end-points in a volume, which is completely within a section and one across the boundary with comparable size. The number of endpoints is an estimate of the stitching quality of the individual datasets. Only endpoints in the intersection of all slices are analysed. b,d,f,h, Average of the tomogram area over all slices for each individual data set as shown in a,c,e,g as a z-projection.
